# Ear-Rational Behavior: A Survey Study of Q-tip (Cotton Swab) Habits and Health Perceptions

**DOI:** 10.7759/cureus.92402

**Published:** 2025-09-15

**Authors:** Brandon Weissman, Shafayath Chowdhury, Michael D Mattin, Francesca Viola, Octavia L Flanagan

**Affiliations:** 1 Otolaryngology, Lake Erie College of Osteopathic Medicine, Elmira, USA; 2 Anesthesiology and Critical Care, Lake Erie College of Osteopathic Medicine, Elmira, USA; 3 Otolaryngology, Ohio University Heritage College of Osteopathic Medicine, Athens, USA; 4 Otolaryngology, University at Buffalo Jacobs School of Medicine and Biomedical Sciences, Buffalo, USA; 5 Primary Care, Lake Erie College of Osteopathic Medicine, Elmira, USA

**Keywords:** cerumen, cotton swabs, ear wax, general otolaryngology, q-tip

## Abstract

Introduction: Q-tips (quality-tips) and cotton swabs have long-standing warnings about their use in the external auditory canal. Despite these warnings, cotton swabs are widely used for these purposes. This study aims to investigate the prevalence, motivations, perceptions of safety, and complications associated with the use of cotton swabs in adults who are engaged on social media.

Methods: A cross-sectional survey was conducted from March to April 2025 using Google Forms (Google, Mountain View, CA, US), distributed via Instagram (Meta Platforms, Inc., Menlo Park, CA, US). Adults aged 18 years or older were eligible, and surveys that were not completed were excluded. The survey consisted of 17-item questions that explored the use of Q-tips.

Results: Of 257 respondents, 229 completed the survey (85.2%, number (n) = 195 aged 18-29 years; 61.1%, n = 140 held a bachelor’s degree). Nearly all participants (95.6%, n = 219) reported using cotton swabs for ear care, primarily to remove cerumen (60.3%, n = 138), with 14.4% (n = 33) reporting daily use. Although 92.6% (n = 212) were aware that medical professionals discourage the use of cotton swabs, 38.4% (n = 88) intended to continue using them, and 38.9% (n = 89) intended to use them occasionally; only 14.4% (n = 33) intended to discontinue their use. Complications were reported by 31.9% (n = 73) of users, most commonly ear discomfort (21.4%, n = 49), cerumen impaction (10.5%, n = 24), and hearing loss (9.2%, n = 21). A small proportion of these individuals sought medical care (8.8%, n = 6). The mean perceived safety score among participants was 3.0 (neutral) on a five-point scale.

Conclusion: The use of Q-tips remains nearly universal in this young, highly educated, and socially media-engaged population, despite widespread awareness of professional warnings. Nearly one-third of users reported complications, yet most planned to continue using the product. These findings underscore a persistent gap between knowledge and behavior, highlighting the need for targeted public health campaigns that not only discourage cotton swab use but also promote safe, practical alternatives for ear care. Social media may be a valuable platform for such interventions.

## Introduction

Q-tips (cotton swabs) were invented in 1923 by Leo Gerstenzang, originally for cleaning his baby’s ears [1]. The Q in Q-tips stands for quality. Q-tips have remained a household staple since their inception. Cotton swabs are often used to remove cerumen from the external ear canal, reduce itchiness/pain, improve hearing, and apply ointments [2].

The external ear has two parts: the auricle (pinna) and the external auditory canal (EAC) (external acoustic meatus). These structures are important for transferring sound to the tympanic membrane [3]. The EAC in adults is usually around 2.5 centimeters (cm) long [3]. The EAC is comprised of a cartilaginous one-third and a bony two-thirds; the cartilaginous portion contains ceruminous and sebaceous glands along with fine hairs [4]. The bony one-third is lined by thinner, more sensitive epithelium.

Cerumen (earwax) is produced by modified apocrine (ceruminous) and sebaceous glands. Secretions from these glands form a lipid-rich mixture that lubricates and waterproofs the external ear canal and tympanic membrane [5]. Cerumen creates a sticky barrier that traps dust, debris, fungal spores, and even small insects. Cerumen also exhibits antimicrobial properties, which are mediated by its slightly acidic potential of hydrogen (pH) as well as lysozymes. The EAC uses cerumen to self-clean the ear; cerumen traps non-physiologic material and migrates out of the ear. This occurs by epithelial migration and jaw movements, which propel the cerumen out of the ear [6].

Cotton swab manufacturers place warning labels on their products. For cotton swabs, this label typically reads, “Do not insert swab into ear canal.” This warning is in place because using cotton swabs in the EAC poses a risk for packing cerumen further in the ear, abrading the ear canal, and possibly perforating the tympanic membrane [2].

Previous research has shown that the use of cotton swabs remains widespread despite the risk of adverse effects. A survey study conducted in patients from an otolaryngologist’s office showed 52% of people used cotton swabs to clean their ears, and 15%-20% of respondents disagreed that cotton swabs can cause complications [1]. Among university students, 65%-92% of those who clean their ears preferentially use cotton swabs, reflecting strong normative influence from family members and peers [2]. Recent parent-focused surveys also reveal persistent beliefs that cotton buds are appropriate for pediatric ear care, underscoring ongoing gaps in public education [7].

Most existing research on cotton swab use has been conducted in clinical and university settings. Social media is becoming a primary source of health-related information among younger populations [8]. This study surveyed adults via Instagram (Meta Platforms, Inc., Menlo Park, CA, US) to determine the prevalence and correlates of cotton swab use for ear cleaning, awareness of professional guidance, reasons for use, perceptions of safety, and self-reported complications.

## Materials and methods

Study design and setting

This study employed a cross-sectional survey design to assess knowledge, attitudes, and practices regarding the use of cotton swabs for ear care. The survey was conducted over one month, from March 11, 2025, to April 4, 2025, using the Google Forms platform (Google, Mountain View, CA, US).

Participants and recruitment

Participants were recruited via a convenience sampling method. The survey link was posted on the investigator’s personal Instagram account, allowing followers and their networks to participate voluntarily. The inclusion criterion was age ≥ 18 years at the time of survey completion. The exclusion criterion was any respondent under 18 years old and anyone who did not fill out all the questions. No compensation was provided.

Survey instrument

The survey consisted of 17 multiple-choice and select-all-that-apply questions designed to capture demographic information (age group, gender, and highest level of education), prior awareness of medical recommendations against cotton swab use, sources of ear care information, frequency and reasons for use, perceived safety, experienced complications, medical care-seeking behavior, and interest in alternative ear-cleaning methods. There was one optional, free-response question that allowed participants to share their comments and beliefs about the use of cotton swabs. (See Appendices for survey questions.) No identifying information was collected on Instagram or Google Forms.

The research questions were developed from the literature review on cotton swab use, clinical knowledge of ear health, and the study’s objectives. Items were selected to measure demographic factors (age, gender, and education) that could influence health behaviors and knowledge, attitudes, and practices regarding Q-tip use. Questions on awareness of medical recommendations, frequency and reasons for use, and perceived safety were included to assess the gap between knowledge and behavior. Complication-related items were designed to capture both self-reported adverse outcomes and subsequent health-seeking behaviors, while final items addressed future intentions and openness to safer alternatives. The survey was designed with multiple-choice and Likert-scale questions to allow for standardized, quantitative analysis, while one open-response option was included to capture additional insights. Because no validated survey exists for cotton swab use, the questionnaire was designed by the investigator. Content validity was established through a literature review of existing literature, but the instrument was not formally validated before deployment.

Data collection and management

Responses were automatically recorded in Google Forms and exported to Microsoft Excel (Microsoft Corp., Redmond, WA, US) for analysis. The data were reviewed for completeness, and ineligible responses (i.e., age < 18 years) were excluded before analysis, as well as those who did not complete the entire survey.

Ethics

This study was reviewed and determined to be exempt by the Lake Erie College of Osteopathic Medicine Institutional Review Board under protocol number 32-118.

## Results

Demographics

Two hundred fifty-seven people attempted to complete the survey; one person was excluded because they were under 18 years of age, and 28 people did not complete the entire survey. Therefore, 229 individuals completed the study and are included in this analysis. The majority of people were aged 18-29 years (195, 85.2%), followed by 30-44 years (22, 9.6%), 45-59 years (7, 3.1%), and 60 years or older (5, 2.2%). The sample was nearly evenly divided by gender, with women comprising 51.5% (118 participants) and men comprising 47.6% (109 participants), while two participants identified as “other” (2, 0.8%). Education level was also recorded, with most respondents holding a bachelor’s degree (140, 61.1%), followed by a master’s degree or other advanced degree (66, 28.8%), some college (17, 7.4%), and a high school diploma or equivalent (6, 2.6%). The demographic data for the study participants are seen in Table 1.

**Table 1:** Demographic data N: number; GED: General Education Development

Category	N (%)
Age group	
18-29 years	195 (85.2%)
30-44 years	22 (9.6%)
45-59 years	7 (3.1%)
≥60 years	5 (2.2)
Gender	
Male	109 (47.6%)
Female	118 (51.5%)
Other	2 (0.8%)
Highest education	
High school/GED	6 (2.6%)
Some college	17 (7.4%)
Bachelor’s degree	140 (61.1%)
Master’s/advanced degree	66 (28.8%)

Awareness and source of information

Most people who took the survey (212, 92.6%) reported that they knew cotton swabs are not recommended for cleaning the ears. Only 14 (6.6%) of participants were unaware of this recommendation, and three (1.3%) were unsure. When asked where they learned about ear cleaning methods, 61 (26.6%) of participants learned from social media or internet sources, 60 (26.2%) from friends or family, 54 (23.6%) from healthcare professionals, 45 (19.7%) had not looked into ear cleaning, and nine (3.9%) from product packaging/instructions.

Prevalence of Q-tip use

Nearly all participants (219, 95.6%) reported using cotton swabs for ear-related purposes. The primary reason was for the removal of cerumen (138, 60.3%), followed by drying ears after swimming or showering (47, 20.5%), and relieving itching (27, 11.8%). A small number reported using cotton swabs for the application of medicine (2, 0.9%), and other reasons (5, 2.2%). Ten (4.4%) of respondents selected non-applicable. Regarding frequency, 33 (14.4%) of participants used cotton swabs daily, 66 (28.8%) used them several times a week, 39 (17%) used them once a week, 32 (14%) used them 1-3 times per month, 47 (20.5%) used them less than once a month, and 12 (5.2%) reported never using them.

Complications and safety

Seventy-three respondents (31.9%) reported at least one complication after using Q-tips (Table 2). Ear pain or discomfort was reported by 49 (21.4% participants), worsened earwax blockage by 24 (10.5%), hearing loss or muffled hearing by 21 (9.2%), ear infection by 11 (4.8%), bleeding by six (2.6%), and dizziness/vertigo by two (0.9%). Of those experiencing complications, 8.8% sought medical attention (15 visited a primary care doctor and five visited an otolaryngologist), 15.7% self-treated, and 75.5% did not seek care. When asked how the complication affected their behavior, 36 (15.7%) participants reduced their use, nine (3.9%) stopped completely, 31 (13.5%) did not change their habit, and 153 selected “not applicable.” As far as perceived safety of cotton swab use, the mean perceived safety score on a 1-5 scale was 3.03 ± 0.9; 111 participants (48.5%) rated the safety as neutral (3), 51 (22.3%) rated it as 4, 43 (18.8%) rated it as 2, 12 (5.2%) rated it as 5, and 12 (5.2%) rated it as 1.

**Table 2:** Complications reported after Q-tip use N: number

Complication	N (%)
No complications	156 (68.1%)
Ear pain or discomfort	49 (21.4%)
Worsened cerumen blockage	24 (10.5%)
Hearing loss/muffled hearing	21 (9.2%)
Ear infection	11 (4.8%)
Bleeding	6 (2.6%)
Dizziness/vertigo	2 (0.9%)
Other (tinnitus, report of friend’s perforation)	2 (0.9%)

Intended future use

After being reminded that medical professionals advise against inserting cotton swabs into the ear canal, 88 (38.4%) participants stated they would continue using them as before, 89 (38.9%) would use them occasionally, 33 (14.4%) intended to discontinue use, and 19 (8.3%) were unsure. Among the 212 respondents who were already aware of professional recommendations, only 27 (12.7%) planned to discontinue use, while 81 planned to use it occasionally, 88 planned to continue, and 16 were unsure.

## Discussion

This cross-sectional survey study yielded a variety of results that shed light on the cotton swab pandemic. It offers a contemporary snapshot of cotton swab use, related complications, and behavioral intentions within a predominantly young adult population. By leveraging Instagram as a recruitment tool for the survey, we were able to capture a broad range of self-reported experiences that extended beyond the clinical setting. The findings from this study warrant further examination within the context of the existing literature, which is unfortunately lacking in both quantity and depth for this particular topic. Nonetheless, the results expose existing gaps between public knowledge, risk perception, and actual ear cleaning practices. The following discussion will compare the results of our study with those of existing studies, explore the sociocultural/behavioral perceptions underlying them, and consider their implications for public health strategy. 

The majority of respondents were aged 18-29 years old (85.2%), which is representative of a population known to be highly digitally connected and actively engaged in social media. Additionally, the sample was well-educated, with approximately 90% of participants holding at least a bachelor’s degree or higher. This suggests a population with widespread access to resources known to improve health literacy. Despite the demographic makeup, 93% of individuals reported using cotton swabs for ear cleaning, with 31.9% experiencing at least one complication.

The most commonly reported complications in this study were ear discomfort (21.4%), wax impaction (10.5%), and hearing loss (9.2%). Each of these complications can have both short-term and long-term implications for otological health. Cerumen impaction frequently causes conductive hearing loss, itching, and a sensation of fullness. These symptoms typically resolve following removal; however, in some cases, they can disrupt audiometry, interfere with proper hearing aid function, and delay the diagnosis of more serious pathologies if not addressed promptly [2]. In addition, impacted wax can also contribute to cognitive decline, particularly in older adults, where evidence shows that removal of impacted wax leads to improvements in both hearing thresholds and cognitive test performance [9].

Beyond hearing loss and cognitive impairment, the use of cotton swabs can also cause trauma to the EAC and tympanic membrane. This increases the risk of perforation, chronic inflammation, and infection, such as otitis externa [10,11]. Inadequate management of chronic otitis externa can lead to persistent pain, discharge, and further hearing impairment, particularly when associated with repetitive epithelial injury or moisture retention [12]. If the tympanic membrane were to become perforated, a sequela would be long-term conductive hearing loss as well as an increased risk of middle ear pathologies or infections [13]. These are just a few examples of the potential threat of cotton swab usage in triggering a cascade of severe outcomes that are more significant than the minor complications reported in this study. This further emphasizes the need for effective awareness and public health education to prevent such disastrous complications.

Comparison with existing literature

This study demonstrated that the use of cotton swabs for ear cleaning is common among young, educated adults on social media, despite widespread awareness of professional warnings. The prevalence of self-ear cleaning in the surveyed population is strikingly consistent with findings from prior studies, which show self-ear cleaning to be nearly universal in some populations. Khan et al. reported that 98% of university students engaged in self-ear cleaning, and the most common method used was the use of cotton buds [2]. Similarly, Olaosun found a self-ear cleaning prevalence of 93.4% among Nigerian young adults, with 85.1% using cotton buds [14]. As mentioned previously, Hobson and Lavy also noted that 53% of otolaryngology clinic patients used cotton buds [1]. The prevalence observed in our sample, with 93% ever use and 51% routine use, is comparable to the statistics found in these studies. This suggests that despite different cultural contexts and decades of health messaging, the practice remains widespread. 

Participants in our study primarily used cotton swabs to remove earwax, which reflected the common belief that cerumen is dirty. However, it is often not known that cerumen acts as a lubricant/barrier, and attempts to remove it can disrupt the epithelium and promote infection [2]. A study by Horton et al. warns that the regular use of cotton-tipped swabs packs wax deeper and leads to cerumen impaction, which can cause complications mentioned previously, such as aural fullness, hearing loss, pain, itching, tinnitus, and otitis externa [15]. Our finding that ear pain, cerumen impaction, and hearing loss were common complications echoes these risks. The 11 participants reporting ear infection and the six reporting bleeding underscore the potential for serious injury. Carniol et al. identified ear canal instrumentation with cotton-tipped applicators as the most frequent cause of traumatic tympanic membrane perforations diagnosed in emergency departments [16]. The study by Hobson and Lavy traced such injuries back to the 1970s [1]. Our survey study provides further evidence that these harms are not simply anomalies.

Interestingly, 92.6% of respondents had previously heard that Q-tips should not be used in the ear. Despite this knowledge, only 14.4% intended to discontinue their use after being reminded, and over two-thirds of participants planned to continue using it occasionally. A similar dissonance between knowledge and behavior was reported by Hobson and Lavy, who found that 15%-20% of patients disagreed that cotton buds can cause infection, wax impaction, or perforation [1]. Another study by Almagribi et al. highlighted the persistent misbelief that earwax removal is necessary [17]. Our data analysis revealed that, even among those aware of the recommendation, 14.4% planned to discontinue use, while 77.3% intended to continue use at the same or reduced frequency. This suggests that awareness alone may be insufficient to change behavior because beliefs about hygiene, sensory satisfaction, and habit may override perceived risk.

The most common sources of information on ear care in our sample were social media, followed by family and friends. In fact, only one-quarter learned of proper ear care methods directly from healthcare professionals. The widespread use of cotton swabs and the high complication rate in our sample could indicate a disregard for public health warnings. Social media can both perpetuate and correct misinformation. Given that cotton swabs have carried warning labels since at least the 1970s and that otolaryngology societies advise against their use, the continued popularity of Q-tips may reflect ineffective communication or contradictory messages [1]. Providers should spend time explaining other validated options for cleaning earwax, according to the American Academy of Otolaryngology-Head and Neck Surgery. Patients should use hydrogen peroxide drops, bulb irrigation, or ear wax softening agents, or have earwax removed by a healthcare provider [18]. Public health campaigns tailored to platforms such as Instagram could emphasize that the ear is self-cleaning, that inserting foreign objects can cause harm, and that professional care should be sought for bothersome wax.

Strengths and limitations

This study utilized a large social media platform to reach a young, educated, and demographically diverse population. The population mainly studied consisted of younger adults, a group that may not be otherwise reached in traditional clinic-based surveys. It captured real-world self-reported practices and outcomes, reflecting behaviors outside clinical confines and offering valuable insight into public health behaviors in a naturalistic context. The large sample size facilitated meaningful subgroup analyses, such as comparisons by age group, gender, education level, and frequency of cotton swab use. Furthermore, the integration of social media as a research tool demonstrates a model that can be replicated for other health behavior studies. This provided a low-cost and time-efficient method for data collection. The questionnaire explored not only usage patterns but also reasons, perceived safety, complications, and behavioral intentions. By including both complication prevalence and behavioral intentions, the study not only identified the scope of the problem but also highlighted opportunities for targeted interventions.

Despite the strengths of this study, the limitations must also be acknowledged. The convenience sample from the investigator’s own network is not representative of the general population and thus limits the generalizability of the findings. Instagram users are predominantly young women, and those interested in participating in the survey may have had particular experiences with cotton swabs. Individuals who are not active on social media, older adults, or those from rural areas are likely underrepresented. Responses are self-reported and subject to recall and social desirability biases. Another limitation is that no clinical verification of reported injuries or side effects was obtained. This means that some respondents may have misattributed unrelated ear symptoms to the use of cotton swabs. The cross-sectional design means no temporal relationship or causality can be inferred between prior education and current cotton swab use. Future research could address these issues by using mixed recruitment strategies, incorporating longitudinal follow-up, and including objective medical assessments of ear health outcomes. Despite these limitations, the findings provide insight into contemporary attitudes toward cotton swab use among social media-engaged adults, while highlighting persistent gaps between knowledge and behavior.

## Conclusions

This survey demonstrates the continued use of cotton swabs despite widespread public awareness of professional discouragement of cotton swab usage. This insight highlights an unaddressed gap between doctor recommendations and actual patient behavior. However, a majority of the participants of the survey expressed interest in learning safer alternatives for ear care, revealing a critical opportunity for additional safe alternatives for ear care. Our findings, consistent with previous studies, underscore that knowledge of risk does not necessarily translate into a change in behavior, particularly within a highly educated, digitally connected young adult demographic. These findings suggest that providers should expand beyond a simple warning against cotton swabs and instead counsel patients on practical alternatives. Social media, a major source of information, may be utilized as a powerful tool for targeted messaging on ear cleaning options. Nearly all respondents reported using cotton swabs for ear cleaning, and almost one-third of users experienced complications ranging from discomfort to hearing loss and infection. Therefore, there is a clear need for targeted, evidence-based public health campaigns that not only inform but also address the underlying motivations for cotton swab use, such as the desire for hygiene and sensory relief, and promote safe, alternative ear care methods. By addressing both behavioral and cultural drivers of ear cleaning practices, providers can reduce preventable complications and promote safer ear health behaviors. Future research should explore the specific psychological and social factors that perpetuate this behavior and evaluate the effectiveness of educational interventions delivered through digital platforms.

## Appendices

Survey Questions

1: Are you over the age of 18? (Select one)

A: Yes

B: No

2: Age Group (Select one)

A: 18-29

B: 30-44

C: 45-59

D: 60+

3: Gender (Select one)

A: Male

B: Female

C: Prefer not to disclose

D: Other (please specify if comfortable)

4: Highest Level of Education Completed (Select one)

A: Some high school or less

B: High school diploma or equivalent (GED)

C: Some college

D: Bachelor’s degree

E: Master’s degree or other advanced degree

F: Prefer not to answer

5: Before today, have you heard that Q-tips (cotton swabs) are NOT recommended for ear cleaning by healthcare professionals? (Select one)

A: Yes, I was aware

B: No, I had not heard this

C: Unsure/cannot remember

6: Where did you primarily learn about proper ear cleaning methods? (Select one)

A: Healthcare professionals (doctor, nurse, pharmacist)

B: Family or friends

C: Social media/internet sources

D: Product packaging/instructions

E: I have not looked into proper ear cleaning methods

7: Have you ever used Q-tips for ear-related purposes? (Select one)

A: Yes, routinely

B: Yes, occasionally

C: No, never

8: If you have used Q-tips for ear-related purposes, what is/was your primary reason? (Select the most relevant one)

A: To clean earwax

B: To relieve itching

C: To dry ears (after showering or swimming)

D: To apply medication in the ear

E: Other 

F: Not applicable/I have never used Q-tips for my ears

9: How frequently do you (or did you) use Q-tips for ear cleaning? (Select one)

A: Daily

B: Several times a week

C: About once a week

D: 1-3 times a month

E: Rarely (less than once a month)

F: Never

10: On a scale of 1-5, how safe do you believe it is to use Q-tips in the ear canal? (Select one)

1 (Very unsafe)

2 (Somewhat unsafe)

3 (Neutral/unsure)

4 (Somewhat safe)

5 (Very safe)

11: Which of the following statements best reflects your thoughts about using Q-tips in the ear? (Select one)

A: I believe it’s generally safe if done gently

B: I believe it’s unsafe, but I do it anyway

C: I believe it’s unsafe and therefore avoid it

D: I was unaware of any concerns or warnings until now

E: Other (please specify)

12: Have you ever experienced any of the following after using a Q-tip in your ear? (Select all that apply)

A: Ear pain or discomfort

B: Bleeding from the ear

C: Worsened earwax blockage

D: Ear infection (otitis externa or otitis media)

E: Dizziness or vertigo

F: Hearing loss or muffled hearing

G: I have not experienced any complications

H: Other (please specify)

13: If you experienced any complication, did you seek medical attention? (Select one)

A: Yes, I went to a primary care doctor

B: Yes, I went to an ENT specialist (otolaryngologist)

C: No, I treated it myself

D: Not applicable/no complications

14: If you experienced complications, how did it affect your Q-tip usage in the future? (Select one)

A: I completely stopped using Q-tips in the ear

B: I reduced how often I use them

C: I did not change my habit

D: Not applicable/no complications

15:After learning (or being reminded) that medical professionals recommend against Q-tip usage in the ear canal, how likely are you to continue or discontinue their use? (Select one)

A: I will discontinue using Q-tips in my ears

B: I may still use them occasionally

C: I will continue using them as before

D: I’m not sure yet

16: Would you be interested in learning about alternative, safer ear cleaning methods? (Select one)

A: Yes, definitely

B: Possibly

C: No, I am not interested

17: Would you like to share anything else about your experience or beliefs regarding Q-tips and ear care? Please do not include any identifiable information here. (Open-ended response - optional.)
